# Non-pharmacological Treatment of Pain: Grand Challenge and Future Opportunities

**DOI:** 10.3389/fpain.2021.696783

**Published:** 2021-05-28

**Authors:** Mary Catherine Bushnell, Eleni Frangos, Nicholas Madian

**Affiliations:** National Center for Complementary and Integrative Health, National Institutes of Health, Bethesda, MD, United States

**Keywords:** pain, complementary medicine, yoga, mediation, exercise, acupuncture

## Introduction

The“Non-pharmacological treatment of pain” section of the *Frontiers in Pain Research* was initiated to reflect the growing appreciation for the role that complementary interventions play in the therapeutic armamentarium for pain management. There are two factors driving this new emphasis. First, there is an increasing number of well-designed clinical trials showing that non-pharmacological modalities have a significant and often enduring efficacy in the management of the pain state with minimal adverse events, whether employed alone or in combination with pharmacological interventions. The real or potential benefits arising from interventions involving mind-body therapeutic modalities take on particular importance in light of the world-wide opiate crisis, which has highlighted the need to develop and understand the mechanisms of novel analgesic treatments. Second, we are beginning to understand the biological mechanisms of non-pharmacological pain treatments, with the development of sophisticated behavioral methodologies, non-invasive imaging techniques, multiple physiological endpoints, and the rigorous adherence to scientific method in both human and pre-clinical studies. Nevertheless, there are many challenges to our obtaining a complete understanding of the efficacy and mechanisms of the myriad non-pharmacological pain treatment modalities.

## Challenges

### Challenges in Terminology

Before addressing other challenges related to non-pharmacological treatments of pain, we must first address the negative connotations of words surrounding many of these methods. When referring to pain treatments that do not involve the use of conventional drugs or surgery, the term “alternative” is often used. If the non-pharmacological treatment is used in conjunction with a drug treatment, such as having a patient exercise, in addition to taking anti-inflammatories for osteoarthritis, the use of exercise can be described as “complementary.” Historically, these terms were used to describe practices which aimed to achieve healing effects, but that lacked biological plausibility and were untested or untestable (1). As such, the terms took on a pejorative meaning to describe methods that had little or no scientific support. Nevertheless, there is a huge difference between untested and untestable, so in 1999, the US Congress created the National Center for Complementary and Alternative Medicine, with the mission to define, through rigorous scientific investigation, the usefulness and safety of complementary and integrative interventions and their roles in improving health and health care. Now, with the opioid crisis, there is an accelerated push around the world to understand the efficacy and safety of non-drug pain treatments, as well as to identify the mechanisms of their effectiveness. However, in order to gain an understanding of efficacy and mechanisms of non-pharmacological pain treatments, we need to first step beyond the idea that anything described as alternative or complementary cannot be evaluated using the scientific method (2). Since the biological basis of a treatment may not always be immediately apparent, we first need well-controlled clinical trials to clearly demonstrate efficacy beyond the placebo effect, and then we need mechanistic studies to identify the biological basis of the effect.

### Challenges in Acceptance of Non-Pharmacological Pain Treatments

Frequently, both patients and clinicians want to be able to treat pain with a pill or with surgery; it is the western medical model (3). Pain treatment that involves more time and effort, such as exercise or meditation, even if proven effective, is difficult to implement in our society (4). One reason for this may be that many non-pharmacological pain treatments are free and easily available, so there is little incentive for industry to promote them. Another obstacle that many chronic-pain patients face is the dynamics of the family system in which they find themselves (5). This system does not play as much of a role when the treatment is something simple like a pill or surgery, but when it is something that requires commitment and lifestyle changes, such as psychotherapy or exercise, family dynamics can form a major obstacle. Furthermore, if it is suggested that their pain can be treated by a mind-body intervention, patients may assume their healthcare practitioners are implying that the pain is “all in their head” and not real, or perhaps even malingered or faked (6), and reject the proposed treatment on this basis. Indeed, the patients may sometimes be correct in these assumptions, given the widespread stigma around pain (7).

### Challenges in Performing Well-Controlled Clinical Trials

One reason that there is a paucity of well-controlled clinical trials for many non-pharmacological pain treatments is that it is difficult to create a parallel placebo or sham manipulation that keeps both patient and clinician blind to the treatment modality. Studies of acupuncture analgesia have found some success using retractable acupuncture needles, but there are still questions about their suitability for subjects who are not naïve to acupuncture (8).

For peripheral and central neural stimulation methods, such as transcutaneous electrical nerve stimulation (TENS) or transcutaneous magnetic stimulation (TMS), a sham control condition is most frequently used, in which the stimulation instrumentation is identical, but no stimulation is actually given during the sham condition. Since the verum stimulation procedures produce a noticeable sensation, sham stimulation is particularly problematic in cross-over designs in which subjects receive both treatments (9, 10).

Even more difficult is to devise adequate control conditions for mind-body therapies, such as yoga or meditation. For yoga, an exercise control group is frequently used (11). However, postures are an integral part of yoga and have been shown to have a major contribution toward neural protective effects of yoga (12). Thus, an exercise control group could mask a major component of the yoga therapeutic effect.

Wait-list groups are frequently used for mind-body interventions, but this does not control for the placebo component of an intervention nor does it allow for double blinding. A better control procedure used for meditation, yoga, cognitive-behavioral therapy, and other mind-body practices involves various types of health education sessions. Although these types of sessions control for time and attention to the subject, they still cannot adequately control for expectations of either the subject or experimenter (11, 13).

The complexity of non-pharmacological treatments is a particular challenge. Interventions such as yoga or cognitive-behavioral therapy are multi-factorial. For example, yoga involves at least three basic components: postures, breathing and meditation. Villemure et al. (12) showed that each of these factors contributes to the protective effect of yoga on age-related gray-matter reduction in the brain. Further, different subjects utilized different components of yoga to different extents, so that the contribution of each factor was not uniform across participants. Thus, a complete understanding of mechanisms of complex mind-body practices would need a separate understanding of each component, as well as the interactive effects of the components with each other—a formidable task.

### Challenges in Understanding Mechanisms of Treatments

In order to fully explore the mechanisms of non-pharmacological pain treatments, we need pre-clinical models that replicate the essential aspects of the clinical therapies. Whereas, preclinical evaluations of pharmacological therapies are straightforward and standard, the concept of having a rodent perform yoga or meditate is almost comical. Certain non-pharmacological analgesic treatments, such as acupuncture or exercise, are amenable to preclinical study. However, even these present unique pitfalls in interpretation. For example, a common method for examining the effects of exercise on pain in rodents involves having the animal run on a treadmill. In order to ensure the running, a procedure is employed in which the animal receives an electric shock when stepping off the treadmill. Such studies find that exercise-related analgesia is opioid mediated, but the possible stress related to impending electric shock confounds the interpretation of these data (14).

## Opportunities

### Development of Novel Control Conditions for Clinical and Preclinical Studies

Over the last decade, studies of acupuncture analgesia have begun to use retractable needles as a control condition. Although the use of these needles does not provide blinding to the investigator, it does provide a control condition for which the subject can be adequately blinded to the procedure (single blind procedure) (15). The continued development of similar “active” sham procedures will lead to an improved ability to separate placebo effects from the active effect of a non-pharmacological manipulation. Active sham stimulation procedures are now being developed for other types of neural stimulation, particularly TMS. The most rigorous sham procedures use the TMS coil for the sham condition, but with an increased distance from the coil to the brain, so that the auditory and visual signals mimic those for the active stimulation, and a sensation on the scalp is felt without activating brain tissue (10).

Finding the best control condition is a challenge in preclinical studies, as well as in human research. Nevertheless, investigators are becoming more aware of the importance of an adequate control condition and are devising novel control conditions. For example, in evaluating the effects of exercise on pain in rodents, one method is to create the same environment as used for exercise, but to preclude the actual exercise, such as providing a locked running wheel that the animal can explore but cannot use for running (16).

### Development of Preclinical Surrogates for Mind-Body Methodologies

One of the most important opportunities for understanding non-pharmacological pain treatments is the development of novel preclinical surrogates for mind-body therapies. Investigators are beginning to devise such surrogates by addressing some individual components of complex methodologies, such as yoga. Human studies of yoga show that postures, breathing, and meditation all contribute to the positive effects of yoga on the brain (12). Yoga postures involve stretching positions that have been simulated in rodents, leading to reduced inflammation, and hypersensitivity after inflammatory injury (17). The meditative aspect of yoga has been partially addressed in rodent studies that compared meditation music to other music and found that meditative music alters addictive behavior in rats (18, 19). Preclinical studies of music therapy are a little-explored avenue in pain research, although there are reports of classical music reducing pain behavior in a rodent bone cancer model (20).

### Use of Virtual Reality to Address Complexity of Mind-Body Treatments

In order to better understand the various components of complex mind-body procedures in human studies, a rich opportunity lies in the use of virtual reality. Studies now show that attentional state, mood and expectation all alter pain perception (21). Mind-body techniques, such as yoga, meditation, tai chi, exercise and music therapy all affect attention, mood and expectation, but to different extents depending on the situation and the individual. Virtual reality has been used extensively to distract patients from pain and has great potential as an analgesic treatment, because of its strong immersive ability (22). Nevertheless, it can also be used as a research tool to separately manipulate aspects of mind-body therapies, including both physical and psychological components.

### Development of Novel Imaging Methodologies Allowing Studies of Brain Mechanisms in Humans and Behaving Rodents

Functional brain imaging methods in humans and rodents continue to develop, increasing both spatial and temporal precision. In humans, the implementation of high resolution 7T MRI, as well as advances in analytical procedures, such as machine learning, can help us better understand the neural basis of non-pharmacological pain treatments. While blood oxygen level dependent (BOLD) functional MRI (fMRI) has provided invaluable information about pain and non-pharmacological pain modulation, it is limited by practical constraints imposed by the scanner environment. Functional near infrared spectroscopy (fNIRS) has emerged as an alternative hemodynamic-based approach that is portable, so that functional brain imaging can occur in studies involving full-body behaviors, such as yoga, meditation or exercise (23). To increase temporal resolution, fNIRS can be combined with EEG. Similarly, other brain imaging methods can be combined (e.g., MRI with Positron Emission Tomography; fMRI with EEG), to access the advantage of each technology. In preclinical studies, calcium imaging of neuronal activity can now be performed in freely moving rodents using miniaturized head mounts (24), allowing real-time studies of the effects of exercise, stretching, environmental enrichment, and other non-pharmacological manipulations.

### Use of Telehealth to Create Larger More Diverse Clinical Trials

A positive outcome of the interruption to research by the COVID-19 pandemic has been the increased use of decentralized clinical trials involving virtual platforms. Building clinical trials around patients in their homes and community through remote visits and monitoring can enhance recruitment and increase the diversity of the subject pool, while also increasing convenience for participants (25). Non-pharmacological manipulations, such as exercise, yoga or meditation, are particularly conducive to remote trialing, as the manipulations can be performed remotely with telehealth technology.

## Conclusions

It is clear from the above discussions that the platform of Non-pharmacological Treatment of Pain represents a broad forum on issues pertinent to the modulation of the pain phenotype in humans and animals. As reviewed in detail elsewhere (https://www.frontiersin.org/journals/pain-research#), *Frontiers in Pain Research* is organized into twelve specialty sections ranging from the basic biology of pain and pain models though pain pharmacology, specific systems and pathologies, neuromodulation, and clinical trialing, with additional focusing on the pediatric and geriatric populations, as well as issues related to the pain states experienced in the veterinary patient. Such specialties reflect organizing foci with which the respective Specialty Chief Editor (SCE) and Associate editors (AE) are assembled, expressing our individual expertise and passions. These specialty components function as a series of linked journals driven by the enthusiasm of their respective SCE/AEs. Not surprisingly, there are major topical overlaps between the 12 specialties. For example, the subject content of Non-pharmacological Treatment of Pain (SCE Catherine Bushnell) intersects with that of the Neuromodulation specialty (SCE Julia Pilitzis). Further, it can be appreciated that a non-pharmacological intervention that focuses on a musculoskeletal or neuropathic pain phenotype will have subject matter that intertwines with the treatment modality or mechanism. The strength of the Pain Research platform is that the review process can draw components of different specialty sections and the attention of different SCEs. It is my aim as the SCE of Non-pharmacological Treatment of Pain to seek integration of topical subject matter and in that manner provide the strongest linkages across the pain specialties.

This is an exciting time for research into non-pharmacological pain therapies. The need for new, improved non-addictive pain treatments has become clear. Both clinicians and patients have the desire to find better ways to treat pain. And, despite the many challenges, both conceptual and technical, in understanding the mechanisms of such treatments, we are now well-positioned to begin a new era of investigation.

## Author Contributions

All authors participated in the inception, writing, commenting of this editorial, and approved the paper.

## Conflict of Interest

The authors declare that the research was conducted in the absence of any commercial or financial relationships that could be construed as a potential conflict of interest.
